# An ecological niche model to predict the geographic distribution of *Haemagogus janthinomys*, Dyar, 1921 a yellow fever and Mayaro virus vector, in South America

**DOI:** 10.1371/journal.pntd.0010564

**Published:** 2022-07-08

**Authors:** Michael Celone, David Brooks Pecor, Alexander Potter, Alec Richardson, James Dunford, Simon Pollett

**Affiliations:** 1 Uniformed Services University of Health Sciences, Bethesda, Maryland, United States of America; 2 Department of Entomology, Walter Reed Army Institute of Research, Silver Spring, Maryland, United States of America; 3 Walter Reed Biosystematics Unit, Suitland, Maryland, United States of America; 4 Infectious Disease Clinical Research Program, Department of Preventive Medicine and Biostatistics, Uniformed Services University of the Health Sciences, Bethesda, Maryland, United States of America; 5 Henry M. Jackson Foundation for the Advancement of Military Medicine, Inc., Bethesda, Maryland, United States of America; The University of Hong Kong, CHINA

## Abstract

Yellow fever virus (YFV) has a long history of impacting human health in South America. Mayaro virus (MAYV) is an emerging arbovirus of public health concern in the Neotropics and its full impact is yet unknown. Both YFV and MAYV are primarily maintained via a sylvatic transmission cycle but can be opportunistically transmitted to humans by the bites of infected forest dwelling *Haemagogus janthinomys* Dyar, 1921. To better understand the potential risk of YFV and MAYV transmission to humans, a more detailed understanding of this vector species’ distribution is critical. This study compiled a comprehensive database of 177 unique *Hg*. *janthinomys* collection sites retrieved from the published literature, digitized museum specimens and publicly accessible mosquito surveillance data. Covariate analysis was performed to optimize a selection of environmental (topographic and bioclimatic) variables associated with predicting habitat suitability, and species distributions modelled across South America using a maximum entropy (MaxEnt) approach. Our results indicate that suitable habitat for *Hg*. *janthinomys* can be found across forested regions of South America including the Atlantic forests and interior Amazon.

## Author summary

Mayaro virus is a neglected tropical disease and there is insufficient evidence to define its geographic range. The mosquito *Haemagogus janthinomys* is a primary vector of Mayaro and its distribution is largely unknown at a sub-country scale. Building compendiums of collection data and creating ecological niche models provides a more precise estimation of vector species potential habitat. Our dataset stands as one of the most expansive existing for collection data of this species combining data published in literature, publicly available data repositories and digitized museum specimen records. Comparing results of niche models with near real time environmental data can give even better predictions of areas where Mayaro virus exposure could occur. The methods and results of this study can be replicated for any disease/vector of interest so long as there is data discoverable through the scientific literature, public repositories, or other civilian and governmental agencies willing to share.

## Introduction

Yellow Fever virus (YFV) is a mosquito-borne flavivirus that causes symptoms including fever, muscle pain, nausea, and fatigue. Although many people recover from initial symptoms of yellow fever, approximately 15 percent of infected patients experience more severe infections including hemorrhage, jaundice, and damage to multiple organ systems [1], with case fatality rates exceeding 40% [2]. Globally, approximately 400 million people are estimated to be at risk of YFV [3]. Although widespread vaccination campaigns have reduced the burden of YFV circulation, several epidemics and epizootics have occurred in South America during the last two decades, primarily in Brazil [4]. In the Americas, YFV predominantly circulates in a sylvatic transmission cycle involving non-human primates and canopy-dwelling mosquitoes of the *Haemagogus (Hg*.*)* subgenus *Haemagogus* [5]. During recent YFV outbreaks in Brazil, *Hg*. *janthinomys* and *Haemagogus* (*Conopostegus*) *leucocelaenus* (Dyar & Shannon, 1924) were implicated as the primary vectors [5]. Contemporary human YFV outbreaks represent spillover events from this sylvatic cycle into the human population [6].

Mayaro virus (MAYV) is a recently emerging arbovirus with a sylvatic transmission cycle throughout Central and South America that occasionally spills over into human populations in Brazil, Bolivia, and Venezuela [7]. While MAYV is not known to be fatal, it can cause non-specific febrile symptoms, and occasionally results in debilitating polyarthritis or polyarthralgia [8]. Although the precise burden of MAYV is unknown, human seroprevalence surveys have detected MAYV circulation in many countries including Peru [9], Suriname [10], Mexico [11], Colombia [12], French Guiana [13], and Haiti [14]. Canopy-dwelling, *Haemagogus janthinomys* Dyar, 1921 is among several mosquito species that are considered important vectors of both YFV and MAYV [15].

*Haemagogus janthinomys* is typically collected in primary rainforest habitats and larvae are typically found in tree holes [15,16]. Adults typically do not fly far from breeding sites in tree canopies where they will feed on non-human primates and other mammals [16]. This species has also been observed to take bloodmeals from up to three different host species in a single ovicycle [17]. Plasticity in host selection, particularly within a single ovicycle could increase chances for zoonotic spill over. *Haemagogus janthinomys will* also descend to the forest floor to feed opportunistically on hosts such as humans [16]. Other *Haemagogus* spp. are known vectors of sylvatic yellow fever, however *Hg*. *janthinomys* appears to have the widest geographic range across central and South America [18]. There does appear to be some plasticity in the feeding behaviors and geographic distribution across central and South America which may indicate this species is actually a complex with cryptic taxa confusing biological observations [19].

A comprehensive understanding of the geographic distribution of *Hg*. *janthinomys* mosquitoes is essential to predicting areas at risk of MAYV and YFV outbreaks. However, it is infeasible to exhaustively survey this species across its entire range, due to site inaccessibility and extensive resource allocation requirements of time, labor, and surveillance equipment. Knowledge of the ecological niche preferences of this species can guide disease surveillance efforts and aid public health authorities in allocating resources for vector control measures. Ecological niche modeling (ENM) techniques have been used extensively to predict the potential range of disease vectors [20], including vectors of Rift Valley fever virus, *Trypanosoma cruzi* [21] and Japanese encephalitis virus [22], among others. Although several modeling studies have used ENM frameworks such as the maximum entropy (MaxEnt) approach to predict the geographic range of the Mayaro [23] and yellow fever viruses [24] and an important YFV vector, *Hg*. *leucocelaenus* [25], there have been very few recent attempts to model the habitat suitability of one of the major vectors, *Hg*. *janthinomys*. This study aims to develop a robust species distribution model of *Hg*. *janthinomys* using a comprehensive dataset of collection records compiled from publicly accessible databases and peer-reviewed literature.

## Methods

### Occurrence and background points

Distribution data for *Hg*. *janthinomys* were compiled from publicly available specimen collection records, archive specimens in the United States National Museum (USNM) mosquito collection, and records reported in peer-reviewed scientific literature. A search of the VectorMap data repository (vectormap.si.edu) yielded both collection locations from USNM specimen records and mosquito surveillance data. Additional collection events were identified from the Global Biodiversity Information Facility (GBIF) database [26] and the NCBI GenBank database [27].

A literature search was conducted using PubMed, Web of Science and Google Scholar. Searches were executed using the keywords “*Haemagogus janthinomys*” combined with each country in South America, including Trinidad and Tobago, for all articles published between 1901 and December 20, 2020. Trinidad and Tobago was included as it is listed as the type locality of *Hg*. *janthinomys*. The search scope was modified to exclude Central American countries after initial searches yielded very few collection records from the literature.

Articles were considered for eligibility based on the following criteria: (i) original research studies on arthropod vectors in South America that described field-collected *Hg*. *janthinomys* adult mosquitoes, larvae, or pupae or original research studies that described the bionomics of *Hg*. *janthinomys*; and (ii) studies that included mappable collection sites (either GPS coordinates or specific named places that can be georeferenced). Articles were not included if they met any of the following exclusion criteria: (i) studies involving only humans; (ii) studies not reporting original data (*e*.*g*., review articles, perspective pieces, editorials, recommendations, and guidelines); (iii) duplicate studies; (iv) laboratory-based vector competence studies or studies involving laboratory-reared mosquitoes; (v) studies that did not provide exact collection site locations; (vi) studies that did not provide information on mosquito identification methods.

All articles were organized using EndNote, and data was abstracted into a Microsoft Excel table. A primary reviewer (MC) independently screened all titles and abstracts to determine articles that could immediately be discarded and articles to be included in the second stage of review. During this second stage of review, full text articles were reviewed to identify candidates for inclusion in the study. A secondary reviewer (AP) examined the screening results to verify the final list of eligible articles. From those studies that met our inclusion criteria, collection data were extracted focusing on all information relevant to preserving the collection event [28]. Locality data for each collection event was georeferenced using the point-radius method [29] and data was standardized using the WRBU/VectorMap Best Practices Guide to Data Management and Reporting [30]. All duplicate coordinates were removed from the dataset. In addition, the *spThin* package in the R statistical software was used to reduce clustering of presence records [31]. A 1-km distance threshold was used to ensure that no more than one presence point occurred within each pixel of the environmental rasters. See the **S1 Table** for a full list of sources, collection dates, and coordinates for each collection location.

A fundamental assumption of ENMs is that species occurrence records are collected through systematic or random sampling (*i*.*e*., unbiased samples) [32], but this assumption is often violated when certain areas are oversampled because they are more easily accessible [33]. This spatial bias can reduce model accuracy because environmental features of these more accessible areas are overrepresented in the model, leading to issues like artificial clustering due to the uneven sampling effort [34] and errors of omission or commission [35]. One solution to correct for sampling bias is the selection of background points with the same selection bias as the presence points [33]. MaxEnt’s default procedure is to select background points at random from the study extent [32]. However, we generated a ‘bias file’ using the *MASS* package in the R statistical software to ensure that the background sampling represented the record density of the occurrence points. As a result, background points were chosen preferentially from areas with a high density of presences. We used MaxEnt’s default setting to select 10,000 background points during the modeling process.

### Variable selection

A total of 32 topographic, climate, and landscape variables were considered for inclusion in the model. The 19 bioclimatic variables from the WorldClim version 2 website were downloaded at a 30 arc-second (~1km) spatial resolution [36]. The Global Multi-resolution Terrain Elevation Data (GMTED) slope and elevation datasets were downloaded from the ESRI Living Atlas of the World database at a 7.5 arc-second (~250m) spatial resolution [37]. The Food and Agricultural Organization’s Digital Soil Map of the World was downloaded at a spatial resolution of 5 arc-minutes (~8km) [38]. Three additional raster layers were created from the initial elevation raster using the flow direction, flow accumulation, and aspect tools from the ESRI ArcGIS Pro Spatial Analyst toolbox [39]. Aspect was transformed to Northness (calculated as cos(Aspect)) using Raster Calculator in ArcGIS Pro [34].

Several variables derived from NASA’s Moderate Resolution Imaging Spectroradiometer (MODIS) remote sensing platform [40] were also considered for our model. This data was provided by the Malaria Atlas Project (https://malariaatlas.org/). Enhanced Vegetation Index (EVI) data and landcover classification data at 2.5 arc-minute (~5km) resolution were accessed from MODIS MCD43D62-68 and MCD12Q1 products, respectively [41,42]. The annual, gap-filled [43] EVI raster layers spanning the years 2000–2020 were used to calculate two synoptic raster layers representing the EVI mean and standard deviation over this time period. In addition, we considered proportional land cover variables for four land cover types that may influence the *Hg*. *janthinomys* distribution: evergreen broadleaf forest, savannas, grasslands, and urban/built up. Annual land cover layers spanning the years 2000–2020 were used to calculate synoptic raster layers representing the mean proportional land cover for each land cover class. Finally, we considered a categorical land cover raster representing the dominant land cover class within each raster grid cell. Land cover classes were based on the International Geosphere–Biosphere Programme (IGBP) classification scheme.

A complete list of the raster layers considered for the model is included in the **S2 Table**. All raster layers were clipped to the extent of South America (15.925°N, 55.983°S, 109.458°W, 28.841°E**)**, re-sampled to a 1000m resolution using bilinear interpolation, and transformed to the WGS 1984 World Mercator projected coordinate system using the Extract by Mask function in ArcGIS Pro. However, it is important to note that resampling of coarser spatial resolution data to the final 1000m spatial resolution did increase the spatial resolution of the variables but did not increase the precision of the data [44]. Country shapefiles were accessed through the geoBoundaries Global Administrative Database [45].

### Covariate significance assessment

A covariate significance assessment was conducted on the sample record collection dataset in order to develop a more refined, statistically robust ENM [46]. Covariate significance was assessed via the t-test and r^2^-maximization criteria at the 95% confidence level (α = 0.05). Number density was used as the dependent variable, and three multiple least-squares regression models were developed and tested to evaluate the linear and quadratic terms of each covariate: 1) a linear-only (LO) model using the linear terms of each covariate; 2) a quadratic-only (QO) model using the quadratic terms of each covariate; and 3) a linear-quadratic (LQ) model that incorporates both the linear and quadratic terms of each covariate. Covariates were tested for correlation/collinearity as part of the covariate significance assessment. Covariates that are directly correlated with other covariates were removed prior to the covariate analysis. For example, bioclim7 was removed due to correlation/collinearity with bioclim5 and bioclim6 (Table 1). As part of the multiple linear and quadratic regression analysis, the correlation coefficient (r) was calculated between each pairwise combination of covariates as an indicator of their degree of correlation (e.g., -1 = high negative correlation; +1 = high positive correlation; 0 = no correlation); and 2-D covariate and correlation matrices were generated that summarize these r values. In addition, a Comparison of Regressions analysis was conducted to assess for significant differences among the correlation coefficient (r), regression slopes (of each covariate in the regression equation), elevation (or regression intercept), and coincidental regression among the regression equations, for each of the regression models (LO, QO, LQ).

**Table 1:** Summary of *Hg*. *janthinomys* presence locations compiled during this study and those incorporated into the model.

Country	Total (n)	Total (%)	Modeled (n)	Modeled (%)
Brazil	122	68.9%	93	65%
French Guiana	20	11.3%	15	10.5%
Colombia	11	6.2%	11	7.6%
Trinidad & Tobago	9	5.1%	9	6.3%
Venezuela	8	4.5%	7	5%
Ecuador	2	1.1%	2	1.4%
Suriname	2	1.1%	2	1.4%
Argentina	1	0.6%	1	0.7%
Bolivia	1	0.6%	1	0.7%
Guyana	1	0.6%	1	0.7%
Peru	1	0.6%	1	0.7%
**Total**	**177**	**100%**	**143**	**100%**

The Multiple Addition (MA) and Multiple Removal (MR) methods were used to sequentially add and remove, respectively, one covariate at a time to and from the developing regression model, and to assess the statistical significance of the covariate addition/removal at each successive step. In the MA process, covariates were sequentially added in the order of highest to lowest significance until all significant (P < 0.05) covariates were added. In the MR process, covariates were sequentially removed in the order of lowest to highest significance until all insignificant (P > 0.05) covariates were removed [46]. A total of six optimal model runs were conducted: three models (LO, QO, LQ) by two sequential methods (MA, MR). A covariate was labeled as significant if at least one of its terms (linear or quadratic) was retained in at least one of the six optimal models (to establish consensus among models).

To assess model variance, the r^2^ and adjusted r^2^ values were calculated at each sequential step to generate plots of these variance measures as a function of the number of covariates terms (N) in the given regression model. Generally, r^2^ increases with an increase in the number of covariate terms (N) in the model, whereas the adjusted r^2^ either increases or decreases. A decrease in adjusted r^2^ with an increase in N reflects the importance of the penalty function (a warning indicator of over-parameterization) offsetting any increase in explained variance obtained by adding the additional covariate term. In any model adjustment, in which covariate terms are added to or removed from the model, tradeoffs exist between r^2^-maximization and covariate significance (ability to explain a significant proportion of variance source, as quantified by r^2^, the correlation coefficient “r”, and P-value). The model exhibiting maximum r^2^ (typically the model with the most covariate terms) is not necessarily the optimal model, especially in cases with relatively small sample sizes, since there’s a potential risk of over-parameterization (low degrees of freedom or difference between sample size and number of model adjustable parameters). Furthermore, in over-parameterized models, adding additional covariate terms does not significantly improve model fit (increase r^2^), and the average variance source explained by each covariate term in the model is usually relatively low, compared to models with fewer but more significant covariate terms.

Bilinear interpolation was used to estimate covariate values at each sample record collection data point by overlaying the covariate raster grids on the sample data points, identifying the 1-km resolution grid-box that each sample data point resides in, converting the geographic coordinates of the sample data point and four corner points of the grid-box to easting-northing, and calculating spatial distances as the basis for interpolation.

### Model settings and performance

The MaxEnt approach to developing ENMs has emerged as one of the most popular techniques for species distribution modeling due to its high predictive accuracy [47] and low sensitivity to sample size [48]. MaxEnt is widely used due to its ability to model complex relationships and interactions between predictors and to avoid overfitting using regularization [48,49]. The MaxEnt technique uses presence-only collection data and a suite of relevant covariates, contrasting environmental conditions at presence points against randomly selected background points [32]. Comparative modeling studies have demonstrated strong performance of the MaxEnt algorithm [50,51]. MaxEnt has also performed well even with a limited sample size [48].

MaxEnt version 3.4.1 was used to generate a distribution map of *Hg*. *janthinomys* habitat suitability using topographic, landscape, and climate variables. An output format of *cloglog* was selected, which returns an estimate between 0 and 1 that represents the habitat suitability within each pixel [52]. The model was run using the *k-fold* cross validation replicated run type with 20 replicates. For this validation procedure, the data set is split into *k* independent subsets (*i*.*e*., folds), and the model is trained on *k-1* subsets and validated using the k^th^ subset [32]. Therefore, each replicate in our model was trained on approximately 90% of the occurrence records and validated using approximately 10% of the occurrence data. In addition, the maximum iterations were set to 10,000 and all other settings were left as their defaults. Features were selected automatically based on the findings of a similar study that reported improved model performance with automatic feature selection versus manual selection [53]. The jackknife test as well as the percent contribution and permutation importance were used to assess the relative importance of each variable in the model. The permutation importance is calculated by randomly permuting the values of each variable on training presence and background data and then reevaluating the model on the permuted data. The resulting drop in training AUC is calculated for each variable, with a greater decrease indicating greater importance of that variable. In order to perform the jackknife procedure, the MaxEnt program runs several models where each variable is omitted in turn, followed by additional models where each variable is used in isolation to predict the species distribution.

The model was run with the significant variables identified from the covariate analysis. The area under the receiver-operating curve (AUC) was used to assess model performance, based on the average AUC across the 20 model replicates. An AUC of 1 suggests that the model perfectly predicts the distribution of the vector while an AUC of 0.5 suggests that the model is not able to predict the distribution better than random chance. We used the AUC of test data (AUC_TEST_) to assess model performance instead of the AUC of training data (AUC_TRAIN_) due to the problems of overfitting associated with the AUC_TRAIN_ statistic [54].

## Results

### *Haemagogus janthinomys* collection data

**Fig 1:**
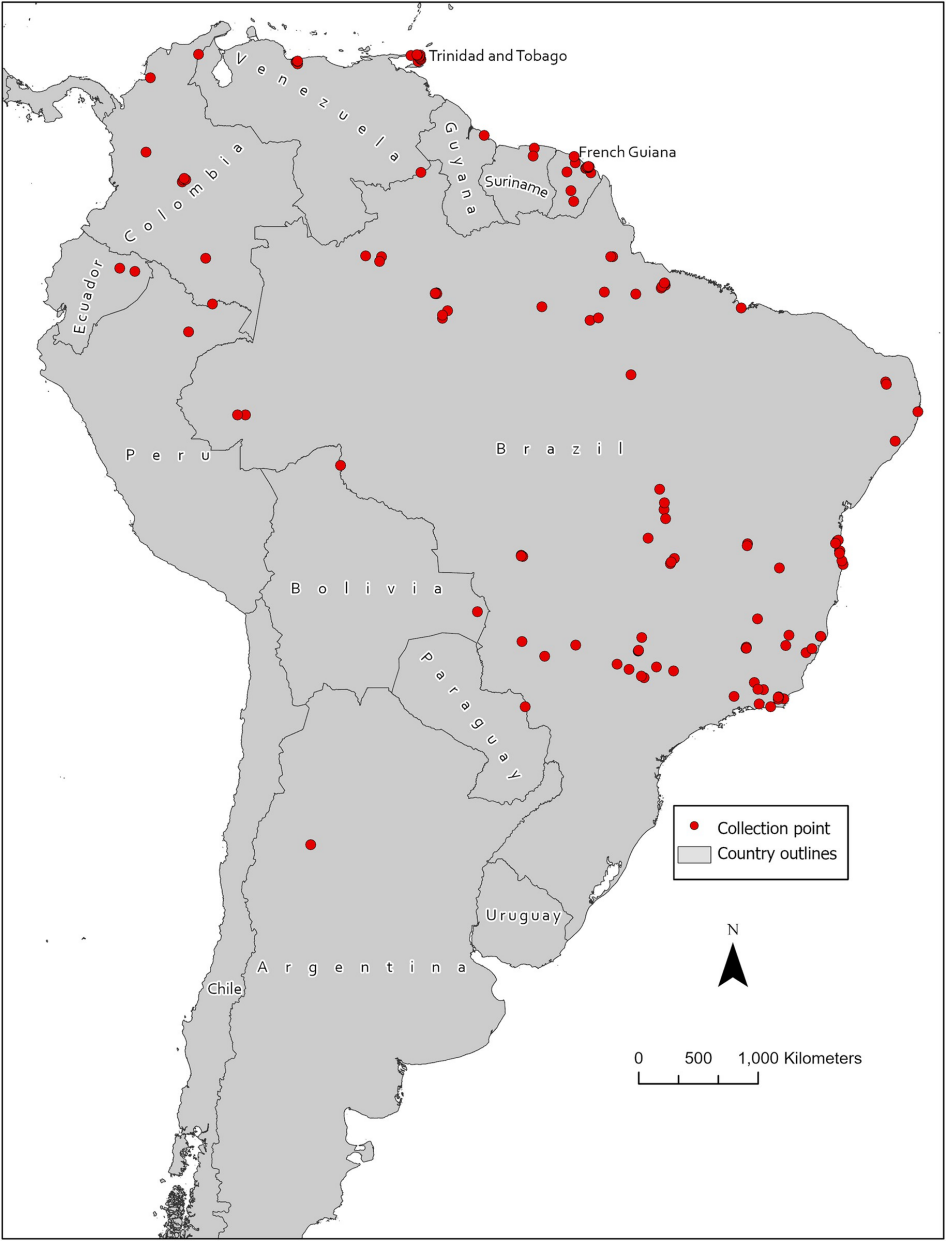
Geographic coordinates of *Hg*. *janthinomys* presence locations compiled during this study*. Base map sourced from Global Administrative Areas (GADM) version 4.0: https://gadm.org/download_country.html.* The Dataset compiled during the study underwent spatial thinning and remaining records were divided into training and testing datasets.

Overall, 177 unique geolocations of verified *Hg*. *janthinomys* presence from 11 countries in South America (including Trinidad and Tobago) were documented, with most records from Brazil (n = 122), French Guiana (n = 20), and Colombia (n = 11) (**see Fig 1 and Table 1**). Within Brazil, the majority of collection events were recorded in the states of Amazonas (n = 20), Rio de Janeiro (n = 16) and Para (n = 14). Overall, the dates of collection ranged from 1935–36 to 2019–2020. The majority of collection events (n = 127) occurred since the year 2000. After thinning the collection events to ensure that no more than one record coincided with each 1-km pixel in the environmental rasters, 34 records were dropped. Therefore, 143 presence points and 10,000 background points were used to develop the ENM. A complete list of collection events is included in the **S1 Table**.

### Covariate selection

Nine continuous covariates were selected for inclusion in the final ENM based on the covariate significance analysis: BIO1 (annual mean temperature), BIO2 (mean diurnal range), BIO5 (maximum temperature of warmest month), BIO6 (minimum temperature of coldest month), BIO9 (mean temperature of driest quarter), BIO14 (precipitation of driest month), BIO15 (precipitation seasonality), BIO19 (precipitation of coldest quarter), and the categorical land cover class (See **Table 2** for variable descriptions). These nine variables were labeled as significant because at least one of their terms (linear or quadratic) were retained in at least one of the six optimal regression models. The BIO15 and land cover variables were retained in four of the optimal models while BIO14 and BIO19 were retained in three of the optimal models. BIO5 was retained in two of the optimal models while BIO1, BIO2, BIO6, and BIO9 were each retained in one optimal model. In addition, a tenth variable, EVI, was included in the model due to the influence of vegetation on the mosquito’s abundance [55–58]. Although this variable was initially discarded based on the results of the covariate significance assessment, we felt that the inclusion of EVI would improve the accuracy of the final model.

**Table 2:** Minimum, maximum, average values, percent contribution, and permutation importance of variables in the *Hg*. *janthinomys* model.

Variable	Description	Min.	Max.	Avg.	Contribution (%)	Permutation (%)
**BIO1**	Average annual temperature, °C	16.8	27.2	24.3	1.4	34.8
**BIO2**	Mean Diurnal Range (Mean of monthly (max temp—min temp)), °C	6.3	14.0	9.5	41	31.6
**BIO5**	Max Temperature of Warmest Month, °C	25.2	34.2	30.8	15.8	10.4
**BIO6**	Min Temperature of Coldest Month, °C	2.9	23.1	17.4	1.1	9.3
**BIO9**	Mean Temperature of Driest Quarter, °C	11.4	28.1	23.5	0.2	0.1
**BIO14**	Precipitation of Driest Month, mm	1.0	231.0	55.3	1.1	1.7
**BIO15**	Precipitation Seasonality (Coefficient of Variation), %	9.9	115.3	56.6	0.4	0.9
**BIO19**	Precipitation of Coldest Quarter, mm	6.0	1312.0	475.5	1.3	2.3
**Enhanced vegetation index**	A measure of canopy greenness	0.2	0.6	0.4	5.7	6.1
**Land cover**	Categorical variable with 17 land cover classes	N/A^a^	N/A	N/A	32.1	2.8

^a^ The most common land cover types at the occurrence points were evergreen broadleaf forest (n = 59), savanna (n = 31), grasslands (n = 19), and urban/built-up (n = 17).

The minimum, maximum, and average values of the nine continuous variables were extracted at each *Hg*. *janthinomys* collection point. The results are presented in **Table 2** along with the percent contribution and permutation importance of each variable according to the MaxEnt model. The average value for the five temperature variables at the *Hg*. *janthinomys* collection points were: 24.3°C (average annual temperature), 9.5°C (mean diurnal range), 30.8°C (max. temperature of warmest month), 17.4°C (min. temperature of coldest month), and 23.5°C (mean temperature of driest quarter). The average value for the three precipitation variables at the *Hg*. *janthinomys* collection points were 55.3mm (precipitation of driest month), 56.6% (precipitation seasonality), and 475.5mm (precipitation of coldest quarter) while the average EVI value was 0.4. Finally, the most common land cover class across the collection points was evergreen broadleaf forest (n = 55).

### Ecological niche model

**Fig 2:**
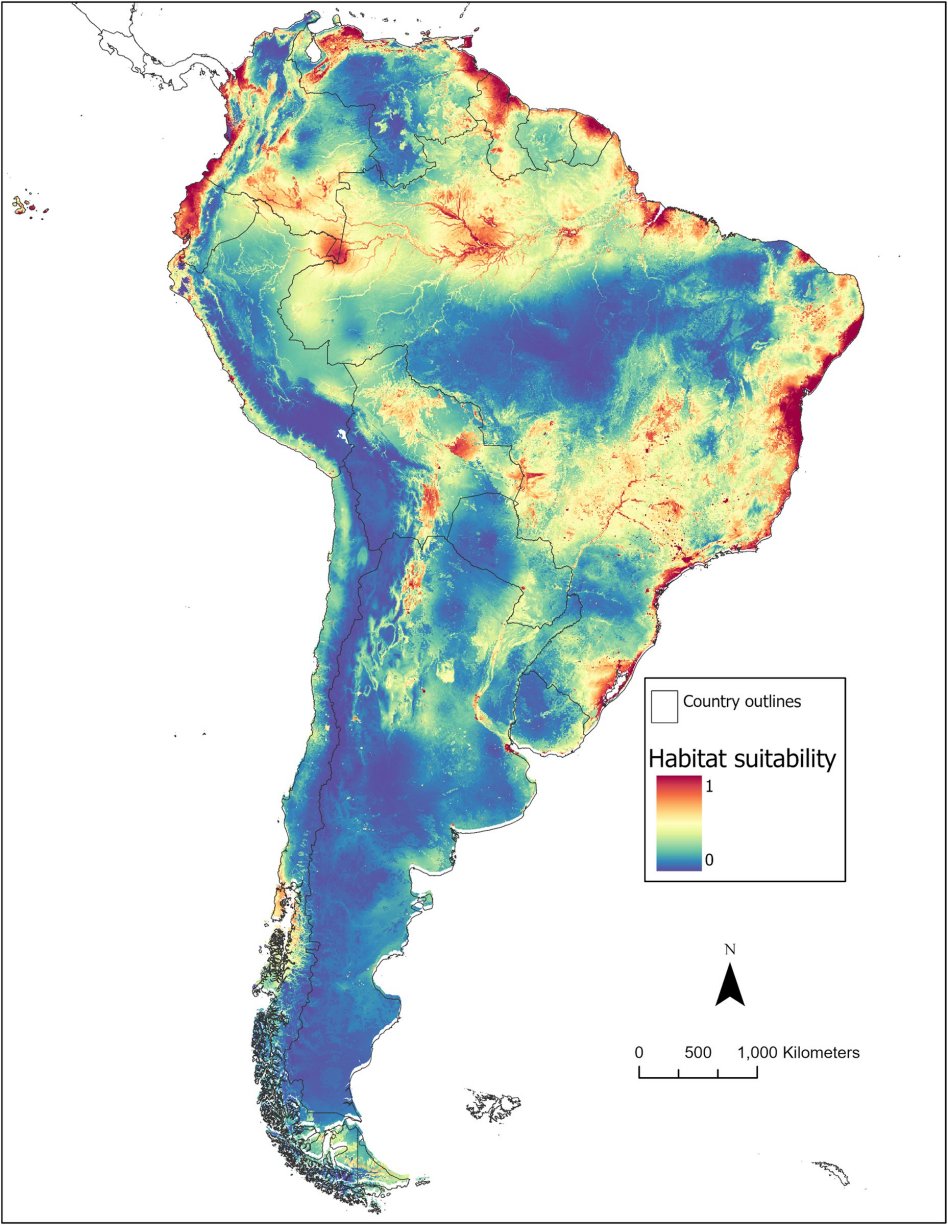
Estimated distribution of the *Hg*. *janthinomys* habitat suitability according to the MaxEnt models. Red represents areas of highest suitability for *Hg*. *janthinomys* while blue represents areas of low suitability. Base map sourced from Global Administrative Areas (GADM) version 4.0: https://gadm.org/download_country.html.

**Fig 3:**
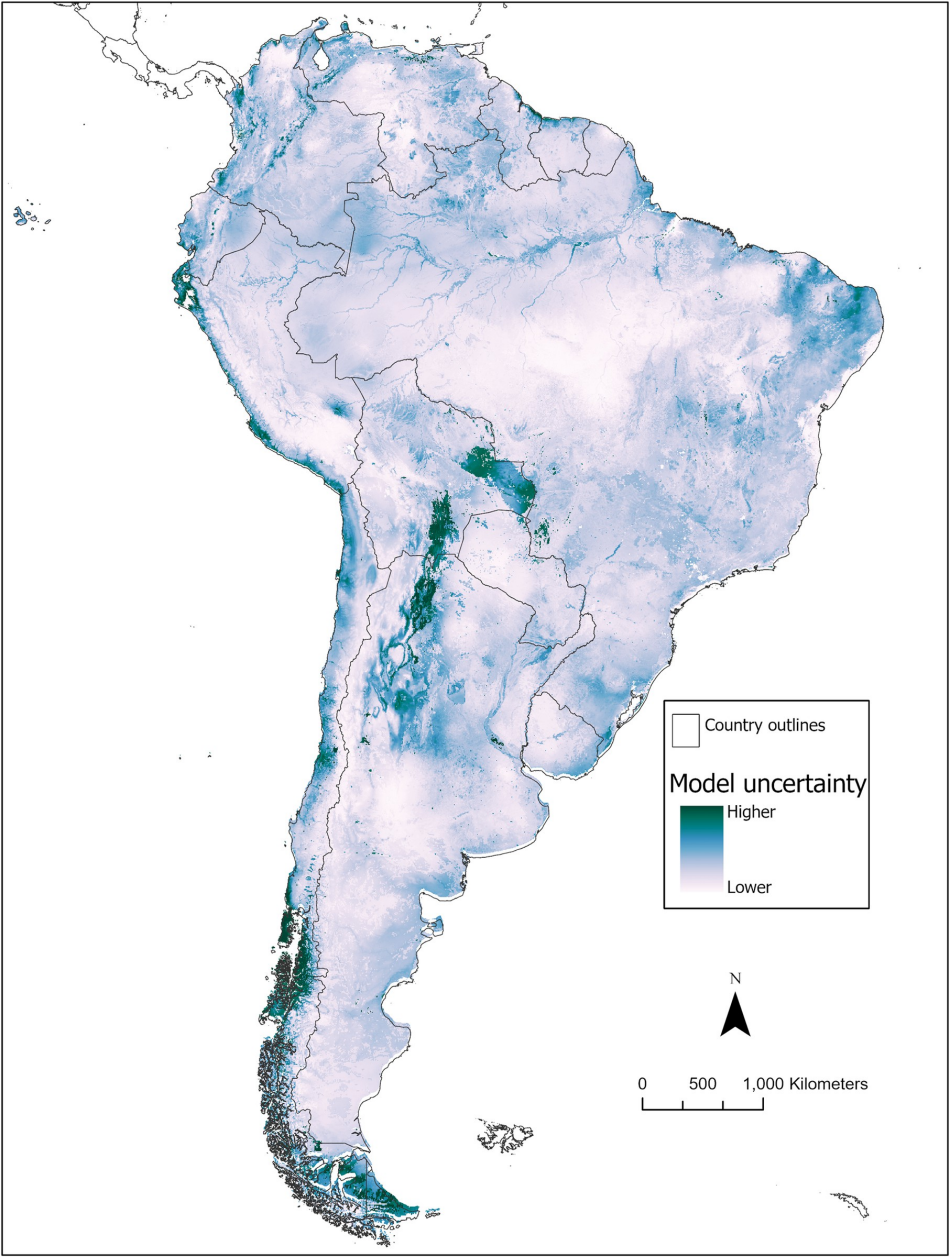
Map of model uncertainty. Estimated uncertainty in spatial prediction of *Hg*. *janthinomys* habitat suitability based on standard deviation for each pixel across the 20 model replicates. Base map sourced from Global Administrative Areas (GADM) version 4.0: https://gadm.org/download_country.html.

The habitat suitability map for *Hg*. *janthinomys* is presented in **Fig 2** and a map of the model uncertainty (i.e., standard deviation) for the 20 replicate runs is presented in **Fig 3**. The suitability map represents the average of the 20 replicate runs incorporating the nine most significant variables identified by the covariate significance assessment and the additional EVI variable that was included in the model. The average area under the receiver operating characteristic curve for testing data (AUC_TEST_) across the 20 model replicates was 0.84 (SD±0.07). The analysis of variable contribution (see **Table 2**) demonstrated that the mean diurnal range contributed the greatest amount of information to the model (41%), followed by land cover (32.1%), and the maximum temperature of the warmest month (15.8%). Similarly, the jackknife tests of both training gain and test gain revealed that these same three variables were the most important variables for developing the ENM. In other words, these variables increased the training/test gain most substantially when used in isolation and decreased the training/test gain most substantially when omitted from the model. In addition, the permutation importance was greatest for average annual temperature (34.8%) followed by mean diurnal range (31.6%).

**Fig 4:**
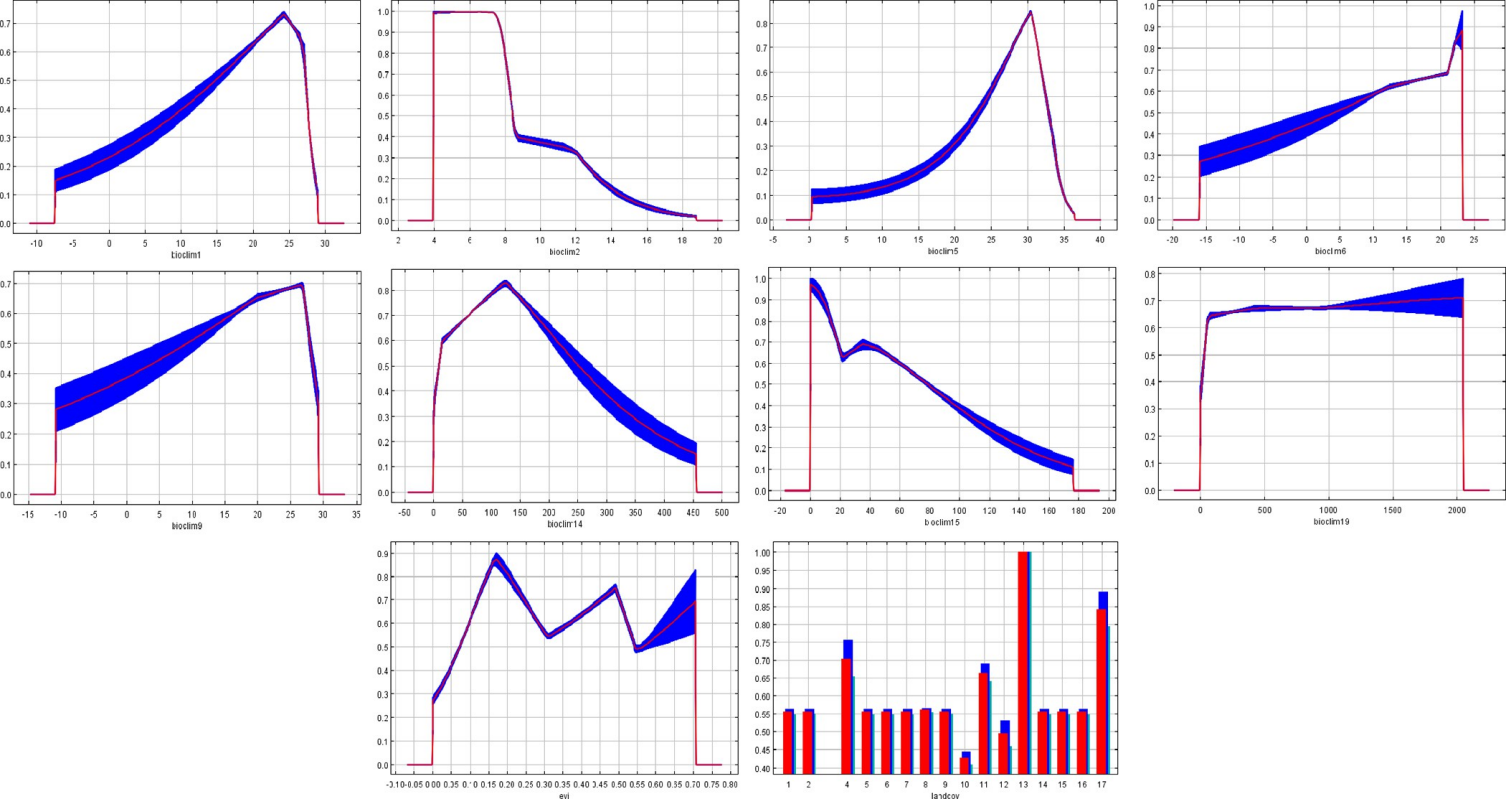
Response curves for the *Hg*. *janthinomys* model. Each curve represents a Maxent model created using only the corresponding variable. The red lines represent the mean response of 20 Maxent runs while blue represents the mean ± 1 standard deviation.

The response curves for each covariate are presented in **Fig 4**. These curves represent the dependence of the predicted suitability on each variable, based on a MaxEnt model created using only the corresponding variable. The response of *Hg*. *janthinomys* to the variable with the greatest percent contribution (mean diurnal range) demonstrated optimal conditions at a lower temperature range, followed by a steep decline as the range increased. The response to the maximum temperature of the warmest month showed an increase in suitability as temperature increased with a peak at 31°C followed by a steep decrease. In addition, the response to the categorical land cover variable demonstrated that the urban/built-up land cover class was highly suitable for *Hg*. *janthinomys* presence. Finally, the response to annual average temperature revealed a steady increase across values that peaked around 24°C, followed by a steep decline. The response curves for the remaining variables are presented in **Fig 4**.

The ENM suggested that the regions with greatest *Hg*. *janthinomys* habitat suitability can be found in the coastal states of Brazil, including Rio Grande do Sul, Santa Catarina, Sao Paulo, etc. High suitability for *Hg*. *janthinomys* presence was also apparent on the Western coast of Colombia (especially in the departments of Choco and Narino) and in several states of Ecuador and Venezuela. The majority of Trinidad and Tobago was found to be highly suitable in addition to the coastal regions of Guyana and French Guiana.

## Discussion

This study has produced a comprehensive database of *Hg*. *janthinomys* collection events as well as a predicted distribution of *Hg*. *janthinomys* habitat suitability. Using the MaxEnt modeling software, we successfully developed a distribution map which provides a contemporary prediction of the mosquito’s potential ecological niche, incorporating the publicly available records of *Hg*. *janthinomys* presence. As an important vector of YF and MAY viruses, it is crucial for health planners to be informed of where this species may contribute to human infections across South America. The model may guide surveillance activities for *Hg*. *janthinomys* by identifying high suitability regions for vector presence.

MaxEnt provides the opportunity to develop ENMs using a relatively small number of collection records. However, the paucity of high-resolution geographical distribution data for *Hg*. *janthinomys* is important to consider when evaluating this model. Our model predicted the potential ecological niche of the *Hg*. *janthinomys* mosquito with an AUC of 0.84, demonstrating several regions of high suitability throughout South America. These findings are consistent with an ecological niche model published in 2010, which found optimal conditions for *Hg*. *janthinomys* along the coast of northeast Brazil and northern Venezuela, based on 78 presence records [18]. Our model included several important variables related to the mosquito’s distribution (e.g., EVI and categorical land cover) that were not included in the previous model [18]. Furthermore, we have included a bias layer to account for sampling bias during the selection of background points. Although our expanded distribution dataset provides a more contemporary prediction of *Hg*. *janthinomys* niche, additional collection data from a wider geographic range is greatly needed.

Our model may serve as an important tool for public health authorities concerned with the spread of MAYV. Although MAYV outbreaks have only occurred sporadically, the Pan American Health Organization (PAHO) has emphasized its growing importance and recommended increased surveillance and enhanced diagnostic capacity [59]. Our model identified areas of high suitability for *Hg*. *janthinomys*, the primary MAYV vector, in the urban coastal region of French Guiana, which aligns with recent epidemiological findings. For example, the World Health Organization (WHO) reported that 11 of 13 confirmed MAYV cases diagnosed in 2020 occurred among residents of the urban coastal areas [60]. Additional locations of MAYV outbreaks in Para, Brazil [61,62] and Portuguesa, Venezuela [63] have also occurred in regions of moderate-to-high suitability for the vector according to our model. During a major outbreak of MAYV in the town of Belterra, Brazil, *Hg*. *janthinomys* mosquitoes were identified as the principal disease vector [64], highlighting the importance of this mosquito in the MAYV transmission cycle.

*Haemagogus janthinomys* has also been identified as an important vector during outbreaks of sylvatic YFV in Brazil over the last several decades [5,65–67]. During the most recent outbreak (2016–18), 20 pools of *Hg*. *janthinomys* were positive for YFV out of 162 pools tested overall and the minimum infection rate (MIR) of *Hg*. *janthinomys* was 34.48, second only to *Hg*. *leucocelaenus* at 34.92 [5]. Therefore, these two mosquito species were identified as the most important sylvatic vectors. The recent YFV outbreaks occurred in areas identified by our model as moderate-to-high suitability for *Hg*. *janthinomys* presence, especially along the coastal areas in the Brazilian states of Sao Paulo, Rio de Janeiro, Espirito Santo, and in the eastern portion of Mato Grosso. *Haemagogus janthinomys* was also implicated as the primary vector during YFV outbreaks in the Atlantic Forest region in the state of Rio de Janeiro during the 1930s-1940s [68]. Therefore, areas predicted as highly suitable for *Hg*. *janthinomys* occurrence may serve as locations of potential disease spillover that could be targeted for increased surveillance and enhanced vector control and disease mitigation efforts.

Prior research has suggested that environmental factors such as temperature and precipitation contribute to *Hg*. *janthinomys* abundance [19]. Several studies have reported that *Hg*. *janthinomys* abundance was substantially higher in the wet season than in the dry season [69–71] and relative humidity was significantly correlated with *Hg*. *janthinomys* abundance [55]. Several variables related to precipitation (the precipitation of the driest month, the precipitation seasonality, and the precipitation of the coldest quarter) were included in our model, although none contributed a substantial amount of information. The greatest suitability for *Hg*. *janthinomys* presence occurred between 0% and 15% precipitation seasonality, suggesting that the mosquito is found in areas of lower variability in rainfall.

The analysis of variable contributions demonstrated that BIO2 (mean diurnal range) had the greatest relative contribution to our model and the greatest training and test gain based on the jackknife test. According to the response curve for BIO2, the ideal diurnal range for mosquito suitability peaks at 6°C followed by a steep decline. Furthermore, the most important variable in our model according to permutation importance was BIO1 (annual average temperature), with peak suitability occurring around 25°C. Several studies have explored the impact of temperature fluctuations on *Aedes* and *Anopheles* mosquito dynamics [72–74], demonstrating that large diurnal temperature range can affect life history traits including larval development time, adult survival, and reproductive output. Our findings also suggest that average temperature and daily temperature fluctuations may impact habitat suitability for *Hg*. *janthinomys*. Peak activity of *Hg janthinomys* has been found to occur during periods of high temperature [69]. In addition, temperature was found to be significantly correlated with *Hg*. *janthinomys* abundance at various canopy heights [55]. However, further research is necessary to better understand the impact of temperature fluctuations on *Hg*. *janthinomys* activity and its ability to transmit pathogens.

The categorical land cover variable also had a large relative contribution to our models. The most common land cover type at the *Hg*. *janthinomys* occurrence points was evergreen broadleaf forest. *Haemagogus janthinomys* is an arboreal species that has recently been detected in many different forest types including mangrove, semi-evergreen seasonal, evergreen seasonal, and young secondary forest [58]. Researchers have captured *Hg*. *janthinomys* mosquitoes in the canopy of the Brazilian rainforest, as high as 16m above the ground [55–57]. The presence of forest cover is therefore an important predictor of the *Hg*. *janthinomys* distribution due to the mosquito’s acrodendrophilic nature. Despite its preference for forest canopies, blood meal analysis has demonstrated that *Hg*. *janthinomys* feeding patterns are diverse, and female mosquitoes may move between the canopy and ground level to collect a blood meal [75]. Although *Hg*. *janthinomys* mosquitoes bite predominantly in the tree canopy, they have demonstrated substantial dispersion, being detected frequently at ground level and in open fields [5,56].

The response of the land cover variable to *Hg*. *janthinomys* suitability identified the urban/built-up land cover class as an important predictor of *Hg*. *janthinomys* presence. Although *Hg*. *janthinomys* mosquitoes are primarily an arboreal mosquito, they have been detected in forest fragments adjacent to major urban areas. For example, Hendy et al., reported the presence of *Hg*. *janthinomys* at an urban park in Manaus, Brazil, within 100m of the forest edge [76] and at another urban park bordering the northern edge of the city [56,76]. Similarly, de Abreu et al., detected *Hg*. *janthinomys* in small forest fragments close to urban areas and in urbanized settlements in recently cut forests [5]. An additional study found *Hg*. *janthinomys* close to agricultural communities in Trinidad [58]. It is evident that forest fragmentation may play an important role in *Hg*. *janthinomys* presence and encourage feeding in peri-domestic or peri-urban environments [77]. Furthermore, *Hg*. *janthinomys* mosquitoes have demonstrated the ability to travel up to 11.5km [78,79]. These findings underscore the importance of *Hg*. *janthinomys* as a potential bridge vector between sylvatic and urban transmission cycles.

Changing patterns of land use/land cover and encroachment into forested areas may increase human exposure to *Hg*. *janthinomys* and to the pathogens they transmit. Several studies have suggested an occupational risk to MAYV infection among rainforest hunters [80] and forest crop-plot workers [81]. In addition, high MAYV seroprevalence has been found in populations residing close to forested areas [82,83]. Communities in close proximity to the forest should therefore be prioritized for vector and pathogen surveillance to determine if MAYV or YFV are circulating in local mosquito populations.

### Limitations

Our study has several limitations that should be considered when interpreting the findings. One major limitation is the sampling bias associated with mosquito collections. Areas of high accessibility, including those in close proximity to roads, are more likely to be sampled, potentially leading to inaccurate models [35]. As a result, the presence records that we have compiled do not represent the complete ecological niche of *Hg*. *janthinomys* but may be biased toward accessible locations. We attempted to correct for sampling bias by including a bias layer during the model-building process, according to techniques proposed by Phillips et al. [33], to ensure selection of background points with the same bias as the presence records. However, it is likely that our results are still impacted by the sampling bias inherent in our data set.

Another limitation inherent in the ecological niche modeling process is the use of a limited set of environmental covariates. While the covariate significance assessment identified insignificant covariates (subsequently removed from the statistically robust ENM) from the complete pool of 32 topographic and bioclimatic covariates, there may be additional covariates not included in this pool that are potentially significant and hence could be incorporated into the ENM. Although these variables play an important role in predicting areas of high suitability for *Hg*. *janthinomys*, other factors such as human population density, socio-economic status, host migration patterns, presence of other mosquito species (in competitive and symbiotic associations for available ecological niches), and intensity of mosquito control and disease mitigation programs can also impact the occurrence probability, habitat suitability, and geographical distributions of *Hg*. *janthinomys*.

An additional limitation is the inability to accurately assess impacts of environmental and topographic variability on *Hg*. *janthinomys* habitat suitability in countries with few *Hg*. *janthinomys* collection points (e.g., Peru). Among the 11 South American countries from which *Hg*. *janthinomys* surveillance data were collected, six countries had two or fewer collection records (collectively 5.6% of the total points used in model), whereas over two-thirds (65%) of the surveillance data used in the model were collected from one country (Brazil), reflecting a highly uneven country-level distribution of surveillance data (Table 1). When data is limited in a geographic area, outliers may carry more weight in the modeling process [48]. Furthermore, model performance may be subject to regional differences, whereby algorithm performance is superior in one region compared to another [48]. This highlights the need for further mosquito sampling across a wider geographic space in order to better characterize the conditions where *Hg*. *janthinomys* occurs.

Another potential limitation is related to the marginal response curve for the land cover variable which demonstrated very high vector habitat suitability in the urban/built-up land cover class. This may be attributed to the relatively narrow range of low urban land cover values at the sample data points. Some inaccuracies may be expected in these predictions, since the model is forced to extrapolate habitat suitability predictions beyond the range of urban land cover values for which the model was calibrated. Sample size was simply insufficient in the range of higher urban land cover for the model to be statistically robust to make realistic predictions of habitat suitability at higher land cover values. In actuality, higher urban land cover may signify lower habitat suitability, particularly since *Hg*. *janthinomys* is known to prefer forested landscapes [58]. Therefore, the species response profile may actually decrease at higher urban land cover values.

Recommendations for future research may include investigating additional mosquito species, identifying under sampled regions via surveillance gap analysis, conducting follow-up surveillance studies in these under sampled regions to reduce sampling bias, incorporating morphological and molecular data on the MAYV and YFV disease pathogens, and running updated models with the enhanced record collection dataset in efforts to further improve the efficacy and statistical robustness of the ecological niche models.

### Disclaimer

Disclaimer: The view(s) expressed in this article are those of the authors and do not necessarily reflect the official policy or position of the Uniformed Services University of the Health Sciences (USU), Henry M. Jackson Foundation for the Advancement of Military Medicine, Inc., the National Institutes of Health or the Department of Health and Human Services, Departments of the Army, Air Force, or Navy, the Department of Defense, or the U.S. Government. The use of trade names in this document does not constitute an official endorsement or approval of the use of such commercial hardware or software. Do not cite this document for advertisement. The publication has been cleared for publication by the Walter Reed Army Institute of Research (WRAIR) and USU.

## Supporting information

S1 Table Coordinates, collection country, collection year, and citations for all collection events. (DOCX)

S2 Table Variables considered for inclusion in the model. (DOCX)

S1 Fig Jackknife results. (TIF)
