# Supplementary material for: An ecological niche model to predict the geographic distribution of Haemagogus janthinomys, Dyar, 1921 a yellow fever and Mayaro virus vector, in South America
Source: PLoS Negl Trop Dis. 2022 Jul 8;16(7):e0010564. doi: 10.1371/journal.pntd.0010564 (PMC9299311; doi:10.1371/journal.pntd.0010564)
Supplement: S1 Table — (DOCX) [file pntd.0010564.s001.docx]

|  | **Coordinates** | | **Collection Year(s)** | **Collection Country** | **Citation** |
| --- | --- | --- | --- | --- | --- |
| ***1*** | -49.79528 | -1.81361 | 1935-36 | Brazil | [1] |
| ***2*** | -61.19843 | 10.58506 | 1941 | Trinidad | [2] |
| ***3*** | -57.78485 | 6.51347 | 1941 | Guyana | [2] |
| ***4*** | -73.61867 | 4.14669 | 1944 | Colombia | [2] |
| ***5*** | -73.64164 | 4.14812 | 1947 | Colombia | [2] |
| ***6*** | -55.20135 | 5.44684 | 1964 | Suriname | [2] |
| ***7*** | -61.2631 | 10.37305 | 1964 | Trinidad | [2] |
| ***8*** | -61.2888 | 10.68057 | 1964 | Trinidad | [2] |
| ***9*** | -61.31597 | 10.72592 | 1964 | Trinidad | [2] |
| ***10*** | -61.11641 | 10.47166 | 1964 | Trinidad | [2] |
| ***11*** | -76.96875 | -0.46999 | 1965 | Ecuador | [2] |
| ***12*** | -73.6868 | 4.07584 | 1965 | Colombia | [2] |
| ***13*** | -73.60555 | 4.18423 | 1965 | Colombia | [2] |
| ***14*** | -73.49741 | 4.21116 | 1965 | Colombia | [2] |
| ***15*** | -73.57838 | 4.26558 | 1965 | Colombia | [2] |
| ***16*** | -52.30125 | 4.86344 | 1965 | French Guiana | [2] |
| ***17*** | -52.25125 | 4.88011 | 1965 | French Guiana | [2] |
| ***18*** | -61.14349 | 10.52606 | 1965 | Trinidad | [2] |
| ***19*** | -61.16068 | 10.70696 | 1965 | Trinidad | [2] |
| ***20*** | -61.19761 | 10.6439 | 1966 | Trinidad | [2] |
| ***21*** | -52.28458 | 4.88011 | 1967 | French Guiana | [2] |
| ***22*** | -53.03462 | 5.41345 | 1967 | French Guiana | [2] |
| ***23*** | -52.41792 | 4.7801 | 1968 | French Guiana | [2] |
| ***24*** | -52.30125 | 4.8301 | 1968 | French Guiana | [2] |
| ***25*** | -52.35125 | 4.88011 | 1968 | French Guiana | [2] |
| ***26*** | -52.30125 | 4.89677 | 1968 | French Guiana | [2] |
| ***27*** | -67.60189 | 10.24685 | 1969 | Venezuela | [2] |
| ***28*** | -67.6594 | 10.32486 | 1969 | Venezuela | [2] |
| ***29*** | -67.68944 | 10.34759 | 1969 | Venezuela | [2] |
| ***30*** | -67.60437 | 10.37887 | 1969 | Venezuela | [2] |
| ***31*** | -67.60433 | 10.38792 | 1969 | Venezuela | [2] |
| ***32*** | -48.40336 | -1.44613 | 1970 | Brazil | [2] |
| ***33*** | -36.63889 | -6.43556 | 1977* | Brazil | [3] |
| ***34*** | -36.595 | -6.55556 | 1977* | Brazil | [3] |
| ***35*** | -54.7312 | -2.4991 | 1978 | Brazil | Genbank |
| ***36*** | -61.625748 | 10.673745 | 1981-82 | Trinidad | [4] |
| ***37*** | -72.815097 | 10.741797 | 1982 | Colombia | [5] |
| ***38*** | -44.24217 | -2.54986 | 1986* | Brazil | [6] |
| ***39*** | -65.33552 | -10.78356 | 1986* | Brazil | [7] |
| ***40*** | -70.35486 | -8.1654 | 1986* | Brazil | [8] |
| ***41*** | -70.76556 | -8.16139 | 1986* | Brazil | [8] |
| ***42*** | -48.414453 | -1.419282 | 1987-88 | Brazil | [9] |
| ***43*** | -48.2433 | -1.3610 | 1991 | Brazil | Genbank |
| ***44*** | -48.46667 | -1.5 | 1994-95 | Brazil | [10] |
| ***45*** | -59.7052 | -2.6998 | 1995 | Brazil | Genbank |
| ***46*** | -48.22000 | -13.53306 | 1996-97 | Brazil | [11] |
| ***47*** | -72.08576 | -2.35092 | 1997 | Colombia | [12] |
| ***48*** | -76.164872 | -0.635613 | 1998 | Ecuador | [2] |
| ***49*** | -72.4466667 | 0.071111 | 1999 | Colombia | [13] |
| ***50*** | -52.2104 | -3.1987 | 1999 | Brazil | Genbank |
| ***51*** | -55.8354444 | -15.4055 | 2000-02 | Brazil | [14] |
| ***52*** | -55.8227222 | -15.4074444 | 2000-02 | Brazil | [14] |
| ***53*** | -55.8348333 | -15.4295833 | 2000-02 | Brazil | [14] |
| ***54*** | -55.83544 | -15.40550 | 2001-02 | Brazil | [15] |
| ***55*** | -55.83483 | -15.42958 | 2001-02 | Brazil | [15] |
| ***56*** | -55.82272 | -15.40744 | 2001-02 | Brazil | [15] |
| ***57*** | -73.34657 | -3.81910 | 2001-02 | Peru | [16] |
| ***58*** | -51.11575 | 0.1373056 | 2002 | Brazil | [17] |
| ***59*** | -51.77453 | -3.08367 | 2004 | Brazil | [18] |
| ***60*** | -48.53917 | -12.02500 | 2004-06 | Brazil | [11] |
| ***61*** | -48.299937 | -12.722924 | 2004-06 | Brazil | [19] |
| ***62*** | -49.14528 | -14.52583 | 2005 | Brazil | [20] |
| ***63*** | -51.45872 | -1.71942 | 2005-06 | Brazil | [21] |
| ***64*** | -51.4587222 | -1.7194167 | 2005-06 | Brazil | [22] |
| ***65*** | -50.059962 | -6.061510 | 2005-07 | Brazil | [23] |
| ***66*** | -49.67938 | -20.20134 | 2006-07 | Brazil | [24] |
| ***67*** | -49.65529 | -20.15661 | 2006-07 | Brazil | [24] |
| ***68*** | -49.64716 | -20.17644 | 2006-07 | Brazil | [24] |
| ***69*** | -48.70739 | -20.98461 | 2006-07 | Brazil | [24] |
| ***70*** | -55.75361 | -15.46639 | 2007 | Brazil | [20] |
| ***71*** | -47.926737 | -15.738265 | 2007-08 | Brazil | [25] |
| ***72*** | -50.78228 | -20.84554 | 2007-08 | Brazil | [24] |
| ***73*** | -50.15897 | -21.10429 | 2007-08 | Brazil | [24] |
| ***74*** | -49.50038 | -21.42567 | 2007-08 | Brazil | [24] |
| ***75*** | -49.36827 | -21.52123 | 2007-08 | Brazil | [24] |
| ***76*** | -47.97147 | -15.81824 | 2007-08 | Brazil | [26] |
| ***77*** | -47.92752 | -15.73719 | 2007-08 | Brazil | [26] |
| ***78*** | -47.77431 | -15.56274 | 2007-08 | Brazil | [26] |
| ***79*** | -42.24162 | -16.04222 | 2007-08 | Brazil | [26] |
| ***80*** | -48.30111 | -13.05444 | 2008 | Brazil | [20] |
| ***81*** | -55.796955 | -19.726460 | 2008 | Brazil | [27] |
| ***82*** | -55.626055 | -22.929018 | 2008 | Brazil | [27] |
| ***83*** | -52.95089 | -19.89787 | 2008 | Brazil | [27] |
| ***84*** | -55.796955 | -19.726460 | 2008 | Brazil | [27] |
| ***85*** | -55.626055 | -22.929018 | 2008 | Brazil | [27] |
| ***86*** | -52.95089 | -19.89787 | 2008 | Brazil | [27] |
| ***87*** | -48.274431 | -1.248649 | 2008 | Brazil | [28] |
| ***88*** | -40.05267 | -19.46704 | 2008-09 | Brazil | [29] |
| ***89*** | -40.07167 | -19.46694 | 2008-09 | Brazil | [30] |
| ***90*** | -43.9397222 | -14.9188889 | 2008-12 | Brazil | [31] |
| ***91*** | -43.92 | -14.81 | 2008-12 | Brazil | [31] |
| ***92*** | -34.946454 | -8.002291 | 2009 | Brazil | [32] |
| ***93*** | -39.19356 | -14.66372 | 2009-14 | Brazil | [33] |
| ***94*** | -39.13739 | -14.61958 | 2009-14 | Brazil | [33] |
| ***95*** | -39.07242 | -15.18203 | 2009-14 | Brazil | [33] |
| ***96*** | -39.05456 | -15.16992 | 2009-14 | Brazil | [33] |
| ***97*** | -64.01083 | 0.19444 | 2010 | Brazil | [34] |
| ***98*** | -63.2894444 | -0.0972222 | 2010 | Brazil | [35] |
| ***99*** | -63.1775 | 0.1530556 | 2010 | Brazil | [35] |
| ***100*** | -47.927627 | -15.737530 | 2010-11 | Brazil | [36] |
| ***101*** | -47.92823 | -15.73852 | 2010-11 | Brazil | [37] |
| ***102*** | -47.92711 | -15.73711 | 2010-11 | Brazil | [37] |
| ***103*** | -43.08417 | -22.09361 | 2011 | Brazil | [38] |
| ***104*** | -44.298157 | -2.602760 | 2011-12 | Brazil | [39] |
| ***105*** | -61.116389 | 4.596944 | 2011-13 | Venezuela | [40] |
| ***106*** | -61.1163889 | 4.5802778 | 2011-13 | Venezuela | [40] |
| ***107*** | -66.90807 | -29.43960 | 2011-15 | Argentina | [41] |
| ***108*** | -51.01383 | 0.15292 | 2012 | Brazil | [42] |
| ***109*** | -51.01383 | 0.15408 | 2012 | Brazil | [42] |
| ***110*** | -75.35139 | 9.52889 | 2012 | Colombia | [43] |
| ***111*** | -61.11278 | 4.59056 | 2013 | Venezuela | [44] |
| ***112*** | -75.592000 | 5.63670 | 2013 | Colombia | [45] |
| ***113*** | -48.2972 | -1.2273 | 2013 | Brazil | [2] |
| ***114*** | -53.075 | 3.05 | 2014 | French Guiana | [2] |
| ***115*** | -52.33 | 4.83 | 2014 | French Guiana | [2] |
| ***116*** | -53.324884 | 4.834089 | 2014 | French Guiana | [2] |
| ***117*** | -53.074507 | 3.050000 | 2014 | French Guiana | [2] |
| ***118*** | -43.379398 | -18.6047 | 2014 | Brazil | [2] |
| ***119*** | -48.297241 | -1.227272 | 2014* | Brazil | [46] |
| ***120*** | -42.2971944 | -22.5528889 | 2014-15 | Brazil | [47] |
| ***121*** | -44.62131 | -22.43114 | 2015 | Brazil | [48] |
| ***122*** | -42.30067 | -22.45425 | 2015 | Brazil | [48] |
| ***123*** | -42.29717 | -22.55317 | 2015 | Brazil | [48] |
| ***124*** | -52.172775 | 4.552363 | 2015 | French Guiana | [2] |
| ***125*** | -53.210740 | 3.627540 | 2015 | French Guiana | [2] |
| ***126*** | -53.21 | 3.63 | 2015 | French Guiana | [2] |
| ***127*** | -52.173 | 4.552 | 2015 | French Guiana | [2] |
| ***128*** | -42.29303 | -22.45392 | 2015-16 | Brazil | [49] |
| ***129*** | -42.28656 | -22.44964 | 2015-16 | Brazil | [49] |
| ***130*** | -42.297712 | -22.454564 | 2015-16 | Brazil | [50] |
| ***131*** | -42.2930278 | -22.4539167 | 2015-16 | Brazil | [51] |
| ***132*** | -42.3026389 | -22.4553889 | 2015-16 | Brazil | [51] |
| ***133*** | -60.314772 | -1.809722 | 2016 | Brazil | [52] |
| ***134*** | -39.075298 | -15.2933 | 2016 | Brazil | [2] |
| ***135*** | -39.2803 | -14.7856 | 2016 | Brazil | [2] |
| ***136*** | -49.486389 | -19.538611 | 2016* | Brazil | [53] |
| ***137*** | -43.311667 | -22.785556 | 2016* | Brazil | [53] |
| ***138*** | -38.947222 | -15.675000 | 2016* | Brazil | [53] |
| ***139*** | -38.882778 | -15.863056 | 2016* | Brazil | [53] |
| ***140*** | -36.132778 | -9.538056 | 2016* | Brazil | [53] |
| ***141*** | -47.801027 | -21.175533 | 2016-17 | Brazil | [54] |
| ***142*** | -42.70774 | -22.92346 | 2016-17 | Brazil | [55] |
| ***143*** | -41.90562 | -19.92002 | 2016-17 | Brazil | [55] |
| ***144*** | -41.7289 | -19.4169 | 2017 | Brazil | Genbank |
| ***145*** | -40.83750 | -20.28556 | 2017 | Brazil | [56] |
| ***146*** | -55.148114 | 5.846099 | 2017 | Suriname | [57] |
| ***147*** | -53.411870 | 4.608840 | 2017 | French Guiana | [2] |
| ***148*** | -52.983104 | 5.077018 | 2017 | French Guiana | [2] |
| ***149*** | -53.412 | 4.609 | 2017 | French Guiana | [2] |
| ***150*** | -52.983 | 5.077 | 2017 | French Guiana | [2] |
| ***151*** | -40.5186111 | -20.0808333 | 2017 | Brazil | [58] |
| ***152*** | -60.2736667 | -1.8037778 | 2017 | Brazil | [59] |
| ***153*** | -60.2739167 | -1.8074167 | 2017 | Brazil | [59] |
| ***154*** | -60.2856111 | -1.7643333 | 2017 | Brazil | [59] |
| ***155*** | -60.3401944 | -1.8118611 | 2017 | Brazil | [59] |
| ***156*** | -60.3231667 | -1.7761667 | 2017 | Brazil | [59] |
| ***157*** | -60.3188611 | -1.8145556 | 2017 | Brazil | [59] |
| ***158*** | -60.2743333 | -1.8065833 | 2017 | Brazil | [59] |
| ***159*** | -60.2739444 | -1.8075 | 2017 | Brazil | [59] |
| ***160*** | -60.2844722 | -1.76375 | 2017 | Brazil | [59] |
| ***161*** | -60.3401389 | -1.8104444 | 2017 | Brazil | [59] |
| ***162*** | -60.3556944 | -1.8043056 | 2017 | Brazil | [59] |
| ***163*** | -60.3590833 | -1.7883056 | 2017 | Brazil | [59] |
| ***164*** | -60.31767 | -1.81733 | 2017-18 | Brazil | [60] |
| ***165*** | -44.0005556 | -20.0680556 | 2017-18 | Brazil | [61] |
| ***166*** | -43.97 | -20.0130556 | 2017-18 | Brazil | [61] |
| ***167*** | -43.9647222 | -20.0725 | 2017-18 | Brazil | [61] |
| ***168*** | -54.598326 | -20.450574 | 2017-18 | Brazil | [62] |
| ***169*** | -43.5591667 | -21.7538889 | 2018 | Brazil | [58] |
| ***170*** | -43.3719444 | -22.0805556 | 2018 | Brazil | [58] |
| ***171*** | -44.229712 | -22.530738 | 2018-19 | Brazil | [63] |
| ***172*** | -44.267396 | -22.550450 | 2018-19 | Brazil | [63] |
| ***173*** | -42.01219 | -22.54388 | 2019 | Brazil | [64] |
| ***174*** | -59.972406 | -3.103798 | 2019 | Brazil | [65] |
| ***175*** | -59.97646 | -2.93776 | 2019-20 | Brazil | [66] |
| ***176*** | -59.96582 | -2.92537 | 2019-20 | Brazil | [66] |
| ***177*** | -59.97069 | -2.92531 | 2019-20 | Brazil | [66] |

*Collection date not provided. Assumed three years before publication date.

References

1. Kumm H, Novis O. Mosquito studies on the Ilha de Marajó, Pará, Brazil. American Journal of Epidemiology. 1938;27(3):498-515.

2. GBIF.org. GBIF Occurrence Download <https://doi.org/10.15468/ikrcdy>. 2020.

3. Xavier SH. Lista das espécies e gêneros de Culicídeos encontrados nos estados do Brasil. VI. Estado do Rio Grande do Norte (Diptera, Culicidae). Mosquito Systematics. 1980;12(3):357.

4. Chadee DD, Tikasingh ES. Observations on the seasonal incidence and diel oviposition periodicity of Haemagogus mosquitoes (Diptera: Culicidae) in Trinidad, W.I.: Part I. Haemagogus janthinomys Dyar. Ann Trop Med Parasitol. 1989;83(5):507-16. Epub 1989/10/01. doi: 10.1080/00034983.1989.11812379. PubMed PMID: 2575884.

5. Morales A, De Carrasquilla CF, de Rodríguez CI, Cura E. Búsqueda de mosquitos de género Haemagogus en el departamento de la Guajira, Colombia, Sur América. Biomedica : revista del Instituto Nacional de Salud. 1984;4(1):25-36.

6. Xavier SH, Mattos SdS. Lista das espécies e gêneros de culicídeos encontrados nos estados do Brasil. IX. Maranhão (Diptera, Culicidae). Acta Amazônica. 1989;19:295-306.

7. Xavier SH, Mattos SdS. Lista das espécies e gêneros de culicídeos encontrados nos Estados do Brasil. VIII. Rondônia (Diptera, Culicidae). Acta Amazônica. 1989;19:285-94.

8. Xavier SH, Mattos SdS, Corrêa IdR. Lista das espécies e gêneros de Culicídeos encontrados nos estados do Brasil. X. Estado do Acre (Diptera, Culicidae). Acta Amazonica. 1989;19:307-17.

9. Dégallier N, Sá Filho GC, da Silva OV, da Rosa AT. Comportamento de pouso sobre partes do corpo humano, em mosquitos da floresta amazonica (Diptera: Culicidae). Boletim do Museu Paraense Emílio Goeldi Nova série Zoologia. 1990;6(2):97-108.

10. Segura MdNO, Monteiro H, Saraiva H, Castro F, Silva O, Vasconcelos PFdC. Entomologic survey of potential of arboviruses in the Combú Island, Belém, Pará State.[Pôster]. 2004.

11. Alencar J, Serra-Friere NM, Marcondes CB, Silva JD, Correa FF, Guimaraes AE. INFLUENCE OF CLIMATIC FACTORS ON THE POPULATION DYNAMICS OF HAEMAGOGUS JANTHINOMYS (DIPTERA: CULICIDAE), A VECTOR OF SYLVATIC YELLOW FEVER. Entomological News. 2010;121(1):45-52. doi: 10.3157/021.121.0109. PubMed PMID: WOS:000289595200007.

12. Ligia Pérez MS, Murcia L, De la Hoz F, Olanos VA, Brochero H, Toro P. La malaria en el Amazonas: conocimientos, prácticas, prevalencia de parasitemia y evaluación entomológica en mayo de 1997. Biomedica : revista del Instituto Nacional de Salud. 1999;19(2):93-102.

13. Molina JA, Hildebrand P, Olano VA, de Hoyos PM, Barreto M, Guhl F. Fauna de insectos hematófagos del sur del Parque Natural Nacional Chiribiquete, Caquetá, Colombia. Biomedica : revista del Instituto Nacional de Salud. 2000;20(4):314-26.

14. Rodrigues FCM. Observações sobre a influência de fatores climáticos nas populações de Haemagogus e Sabethes (Diptera: Culicidae) vetores de febre amarela silvestre. Instituto Oswaldo Cruz, Fundação Oswaldo Cruz, Rio de Janeiro. Available from: <https://www.arca.fiocruz.br/handle/icict/34805> 2016.

15. Alencar J, De Mello CF, Morone F, Albuquerque HG, Sirra-Freire NM, Gleiser RM, et al. DISTRIBUTION OF HAEMAGOGUS AND SABETHES SPECIES IN RELATION TO FOREST COVER AND CLIMATIC FACTORS IN THE CHAPADA DOS GUIMARAES NATIONAL PARK, STATE OF MATO GROSSO, BRAZIL. Journal of the American Mosquito Control Association. 2018;34(2):85-92. doi: 10.2987/18-6739.1. PubMed PMID: WOS:000440151100001.

16. Turell MJ, Gozalo AS, Guevara C, Schoeler GB, Carbajal F, López-Sifuentes VM, et al. Lack of Evidence of Sylvatic Transmission of Dengue Viruses in the Amazon Rainforest Near Iquitos, Peru. Vector-Borne and Zoonotic Diseases. 2019;19(9):685-9. doi: 10.1089/vbz.2018.2408.

17. Souto RNP. Inventário da fauna culicidiana (Diptera: Culicidae) na ressacas do Curralinho e da Lagoa dos Índios. Diagnóstico das ressacas do Estado do Amapá: bacias do Igarapé da Fortaleza e Rio Curiaú CPQAP/IEPA and DGEO/SEMA, Macapá. 2003:63-72.

18. Nunes Neto JP, Monteiro H, Segura MdNO, Cantuária P, Saraiva H, Silva O, et al. Criação de Haemagogus (Haemagogus) janthinomys (Díptera: Culicidae) utilizando xarope de guaraná 50%. 2005.

19. Silva JD, Pacheco JB, Alencar J, Guimaraes AE. Biodiversity and influence of climatic factors on mosquitoes (Diptera: Culicidae) around the Peixe Angical hydroelectric scheme in the state of Tocantins, Brazil. Memorias Do Instituto Oswaldo Cruz. 2010;105(2):155-62. doi: 10.1590/s0074-02762010000200008. PubMed PMID: WOS:000277152900008.

20. Alencar J, Gil-Santana HR, de Oliveira RdFN, Dégallier N, Guimarães AÉ. Natural breeding sites for Haemagogus mosquitoes (Diptera, Culicidae) in Brazil. Entomological News. 2010;121(4):393-6.

21. Confalonieri UE, Costa Neto C. Diversity of mosquito vectors (Diptera: culicidae) in caxiuana, para, Brazil. Interdisciplinary perspectives on infectious diseases. 2012;2012.

22. Pinto CS, Confalonieri UE, Mascarenhas BM. Ecology of Haemagogus sp. and Sabethes sp. (Diptera: Culicidae) in relation to the microclimates of the Caxiuanã National Forest, Pará, Brazil. Mem Inst Oswaldo Cruz. 2009;104(4):592-8. Epub 2009/09/02. doi: 10.1590/s0074-02762009000400010. PubMed PMID: 19722082.

23. Monteiro H. Avaliação da diversidade de insetos hematófagos da subordem Nematocera e de vertebrados silvestres: transmissão de arbovírus na área de influência do Projeto Salobo, Carajás, Pará: Universidade Federal do Pará, Instituto de Ciências Biológicas. Programa de Pós-Graduação em Biologia de Agentes Infecciosos e Parasitários. Available at: <http://repositorio.ufpa.br/jspui/handle/2011/4748>; 2009.

24. Mucci LF, Júnior RP, de Paula MB, Scandar SA, Pacchioni ML, Fernandes A, et al. Feeding habits of mosquitoes (Diptera: Culicidae) in an area of sylvatic transmission of yellow fever in the state of São Paulo, Brazil. J Venom Anim Toxins Incl Trop Dis. 2015;21:6. Epub 2015/03/27. doi: 10.1186/s40409-015-0005-z. PubMed PMID: 25810711; PubMed Central PMCID: PMCPMC4373060.

25. dos Santos J, Takashi M, Cavalcante K, Steinke E. Culicídeos encontrados em áreas próximas a Piscina Velha do Parque Nacional de Brasília (DF). Hygeia-Revista Brasileira de Geografia Médica e da Saúde. 2008;4(6):157-62.

26. Obara MT, Monteiro H, Paula MBd, Gomes AdC, Yoshizawa MAC, Lira AR, et al. Infecção natural de Haemagogus janthinomys e Haemagogus leucocelaenus pelo vírus da febre amarela no Distrito Federal, Brasil, 2007-2008. Epidemiologia e Serviços de Saúde. 2012;21(3):457-63.

27. de Almeida PS, da Silva JO, Ramos EP, Batista PM, Faccenda O, de Paula MB, et al. Vector aspects in risk areas for sylvatic yellow fever in the state of Mato Grosso do Sul, Brazil. Revista de Patologia Tropical/Journal of Tropical Pathology. 2016;45(4):398-411.

28. Azevedo RS, Silva EV, Carvalho VL, Rodrigues SG, Neto JPN, Monteiro HA, et al. Mayaro fever virus, Brazilian amazon. Emerg Infect Dis. 2009;15(11):1830. doi: 10.3201/eid1511.090461.

29. Rezende HR, Virgens TMd, Liberato MA, Valente FI, Fernandes A, Urbinatti PR. Aspectos ecológicos de culicídeos imaturos em larvitrampas de floresta e ambiente antrópico adjacente no Município de Linhares, Espírito Santo, Brasil. Epidemiologia e Serviços de Saúde. 2011;20(3):385-91.

30. Virgens TMd, Rezende HR, Pinto IS, Falqueto A. Fauna of mosquitoes (Diptera: Culicidae) in Goytacazes National Forest and surrounding area, State of Espírito Santo, Southeastern Brazil. Biota Neotropica. 2018;18(1).

31. Santos CF, Silva AC, Rodrigues RA, JESUS JSRd, Borges MAZ. Inventory of mosquitoes (Diptera: Culicidae) in conservation units in Brazilian tropical dry forests. Revista do Instituto de Medicina Tropical de Sao Paulo. 2015;57(3):227-32.

32. Aragao NC, Muller GA, Balbino VQ, Costa CRL, Figueiredo CS, Alencar J, et al. A list of mosquito species of the Brazilian State of Pernambuco, including the first report of Haemagogus janthinomys (Diptera: Culicidae), yellow fever vector and 14 other species (Diptera: Culicidae). Revista Da Sociedade Brasileira De Medicina Tropical. 2010;43(4):458-9. doi: 10.1590/s0037-86822010000400024. PubMed PMID: WOS:000281426100024.

33. Catenacci LS, Nunes-Neto J, Deem SL, Palmer JL, Travassos-da Rosa ES, Tello JS. Diversity patterns of hematophagous insects in Atlantic forest fragments and human-modified areas of southern Bahia, Brazil. Journal of Vector Ecology. 2018;43(2):293-304. doi: 10.1111/jvec.12313. PubMed PMID: WOS:000449478600010.

34. Hutchings RSG, Hutchings RW, Menezes IS, Motta MD, Sallum MAM. Mosquitoes (Diptera: Culicidae) From the Northwestern Brazilian Amazon: Padauari River. Journal of Medical Entomology. 2016;53(6):1330-47. doi: 10.1093/jme/tjw101. PubMed PMID: WOS:000390217500011.

35. Hutchings RSG, Hutchings RW, Menezes IS, Motta MD, Sallum MAM. Mosquitoes (Diptera: Culicidae) From the Northwestern Brazilian Amazon: Araca River. Journal of Medical Entomology. 2018;55(5):1188-209. doi: 10.1093/jme/tjy065. PubMed PMID: WOS:000456130900015.

36. Lira-Vieira AR, Gurgel-Gonçalves R, Moreira IM, Yoshizawa MA, Coutinho ML, Prado PS, et al. Ecological aspects of mosquitoes (Diptera: Culicidae) in the gallery forest of Brasília National Park, Brazil, with an emphasis on potential vectors of yellow fever. Rev Soc Bras Med Trop. 2013;46(5):566-74. Epub 2013/10/22. doi: 10.1590/0037-8682-0136-2013. PubMed PMID: 24142367.

37. Vieira ARL. Distribuição de espécies de Culicídeos (Diptera, Culicidae) em mata de galeria no Parque Nacional de Brasília, DF: M.S.c Thesis. Universidade de Brasília. Available from: <http://bdtd.ibict.br/vufind/Record/UNB_4b77b4f2811e191b4cc75204f3b57a5b>; 2012.

38. Alencar J, Gleiser RM, Morone F, de Mello CF, Silva JD, Serra-Freire NM, et al. A comparative study of the effect of multiple immersions on Aedini (Diptera: Culicidae) mosquito eggs with emphasis on sylvan vectors of yellow fever virus. Memorias Do Instituto Oswaldo Cruz. 2014;109(1):114-7. doi: 10.1590/0074-0276130168. PubMed PMID: WOS:000333038700015.

39. Pereira ACN. Ecologia de mosquitos (Diptera: Culicidae) na Área de Proteção Ambiental do Maracanã, São Luís, Maranhão, Brasil. Fundação Oswaldo Cruz, Instituto Oswaldo Cruz, Rio de Janeiro, RJ. Available at: <https://www.arca.fiocruz.br/handle/icict/182412016>.

40. Berti J, Guzmán H, Estrada Y, Ramírez R. New records of mosquitoes (Diptera: Culicidae) from Bolívar State in South Eastern Venezuela, with 27 new species for the state and 5 of them new in the country. Frontiers in public health. 2015;2:268.

41. Linares M, Stein M, Laurito M, Rossi G, Visintín A, Almirón W. New mosquito records (Diptera: Culicidae) from northwestern Argentina. Check List. 2016;12:1.

42. Cantuaria MF. Ecologia de culicídeos (Diptera: Culicidae) da área de proteção ambiental do rio Curiaú, Macapá, Amapá. Macapá Programa de Pós Graduação em Biodiversidade Tropical. Available from: <https://www2.unifap.br/ppgbio/files/2010/05/disserta%C3%A7%C3%A3o_Maryele_Ferreira.pdf>: Universidade Federal do Amapa; 2012

43. Bustamante SC. Identificación morfológica y molecular de especies del género haemagogus (diptera: culicidae) en la región caribe colombiana: Universidad de Sucre, Colombia. Available from: <https://repositorio.unisucre.edu.co/handle/001/970>; 2019.

44. Berti J, Estrada Y, Guzmán H, Ramirez R, Pérez E. Nuevos registros geográficos para Haemagogus anastasionis Dyar, 1921 y Haemagogus janthinomys Dyar, 1921 (Diptera: Culicidae) en Venezuela. Entomotropica. 2014;29(2):121-4.

45. Rozo-Lopez P, Mengual X. Mosquito species (Diptera, Culicidae) in three ecosystems from the Colombian Andes: identification through DNA barcoding and adult morphology. ZooKeys. 2015;(513):39.

46. Lemos PD, Monteiro HA, Castro FC, Lima CP, Silva DE, Vasconcelos JM, et al. Characterization of mitochondrial genome of Haemagogus janthinomys (Diptera: Culicidae). Mitochondrial DNA Part A, DNA mapping, sequencing, and analysis. 2017;28(1):50-1. Epub 2015/12/29. doi: 10.3109/19401736.2015.1110793. PubMed PMID: 26709451.

47. Alencar J, de Mello CF, Rodríguez‐Planes L, dos Santos Silva J, Gil‐Santana HR, Bastos AQ, et al. Ecosystem diversity of mosquitoes (Diptera: Culicidae) in a remnant of Atlantic Forest, Rio de Janeiro state, Brazil. Austral Entomology. 2020.

48. Alencar J, Mello CF, Barbosa LS, Gil-Santana HR, Maia Dde A, Marcondes CB, et al. Diversity of yellow fever mosquito vectors in the Atlantic Forest of Rio de Janeiro, Brazil. Rev Soc Bras Med Trop. 2016;49(3):351-6. Epub 2016/07/08. doi: 10.1590/0037-8682-0438-2015. PubMed PMID: 27384833.

49. Silva SOF, Ferreira de Mello C, Figueiró R, de Aguiar Maia D, Alencar J. Distribution of the Mosquito Communities (Diptera: Culicidae) in Oviposition Traps Introduced into the Atlantic Forest in the State of Rio de Janeiro, Brazil. Vector Borne Zoonotic Dis. 2018;18(4):214-21. Epub 2018/03/30. doi: 10.1089/vbz.2017.2222. PubMed PMID: 29595406; PubMed Central PMCID: PMCPMC5878547.

50. Silva SOF, de Mello CF, Gleiser RM, Oliveira AA, Maia DD, Alencar J. Evaluation of Multiple Immersion Effects on Eggs From Haemagogus leucocelaenus, Haemagogus janthinomys, and Aedes albopictus (Diptera: Culicidae) Under Experimental Conditions. Journal of Medical Entomology. 2018;55(5):1093-7. doi: 10.1093/jme/tjy080. PubMed PMID: WOS:000456130900004.

51. Alencar J, de Mello CF, Guimarães AÉ, de Aguiar Maia D, de Queiroz Balbino V, de Souza Freitas MT, et al. The first detection of a population of Aedes aegypti in the Atlantic Forest in the state of Rio de Janeiro, Brazil. Tropical Zoology. 2020;33(2).

52. Silva JWP. Aspectos ecológicos de vetores putativos do Vírus Mayaro e Vírus Oropuche em estratificação vertical e horizontal em ambientes florestais e antropizados em uma comunidade rural no Amazonas [M.Sc. Thesis]. Manaus, AM: Oswaldo Cruz Foundation, Instituto Leônidas and Maria Deane; 2017. Available from: <https://www.arca.fiocruz.br/handle/icict/23337>.

53. Silva SOF, Fuente ALC, Mello CF, Alencar J. Morphological differentiation between seven Brazilian populations of Haemagogus capricornii and Hg. janthinomys (Diptera: Culicidae) using geometric morphometry of the wings. Rev Soc Bras Med Trop. 2019;52:e20180106. Epub 2019/01/18. doi: 10.1590/0037-8682-0106-2018. PubMed PMID: 30652787.

54. Cunha MS, da Costa AC, de Azevedo Fernandes NCC, Guerra JM, Dos Santos FCP, Nogueira JS, et al. Epizootics due to yellow fever virus in São Paulo State, Brazil: viral dissemination to new areas (2016–2017). Scientific reports. 2019;9(1):1-13.

55. Moutailler S, Yousfi L, Mousson L, Devillers E, Vazeille M, Vega-Rúa A, et al. A new high-throughput tool to screen mosquito-borne viruses in Zika virus endemic/epidemic areas. Viruses. 2019;11(10):904.

56. Gómez MM, Abreu FVS, Santos A, Mello IS, Santos MP, Ribeiro IP, et al. Genomic and structural features of the yellow fever virus from the 2016-2017 Brazilian outbreak. The Journal of general virology. 2018;99(4):536-48. Epub 2018/02/23. doi: 10.1099/jgv.0.001033. PubMed PMID: 29469689.

57. Visser TM, De Cock MP, Hiwat H, Wongsokarijo M, Verhulst NO, Koenraadt CJ. Optimisation and field validation of odour-baited traps for surveillance of Aedes aegypti adults in Paramaribo, Suriname. Parasites & vectors. 2020;13(1):1-14.

58. Delatorre E, de Abreu FVS, Ribeiro IP, Gómez MM, Dos Santos AAC, Ferreira-de-Brito A, et al. Distinct YFV Lineages Co-circulated in the Central-Western and Southeastern Brazilian Regions From 2015 to 2018. Frontiers in microbiology. 2019;10:1079. Epub 2019/06/11. doi: 10.3389/fmicb.2019.01079. PubMed PMID: 31178835; PubMed Central PMCID: PMCPMC6543907.

59. Almeida JF, Belchior HCM, Ríos-Velásquez CM, Pessoa FAC. Diversity of mosquitoes (Diptera: Culicidae) collected in different types of larvitraps in an Amazon rural settlement. PloS One. 2020;15(10):e0235726.

60. Almeida JF. Evidências de Sinatropização de mosquitos (DIPTERA: Culicidae) em um assentamento rural na Amazônia brasileira [M.Sc. Thesis]: Instituto Leônidas e Maria Deane, Fundação Oswaldo Cruz, Manaus, 2018. Available from: <https://www.arca.fiocruz.br/handle/icict/31084>; 2018.

61. Pinheiro GG, Rocha MN, de Oliveira MA, Moreira LA, Andrade Filho JD. Detection of Yellow Fever Virus in Sylvatic Mosquitoes during Disease Outbreaks of 2017⁻2018 in Minas Gerais State, Brazil. Insects. 2019;10(5). Epub 2019/05/15. doi: 10.3390/insects10050136. PubMed PMID: 31083286; PubMed Central PMCID: PMCPMC6572267.

62. Pauvolid-Corrêa A, Gonçalves Dias H, Marina Siqueira Maia L, Porfírio G, Oliveira Morgado T, Sabino-Santos G, et al. Zika Virus Surveillance at the Human–Animal Interface in West-Central Brazil, 2017–2018. Viruses. 2019;11(12):1164.

63. Rodrigues FdC. O efeito de uma interface de paisagem florestal-urbana sobre a distribuição de mosquitos vetores de arbovírus e vigilância entomológica no município de Barra Mansa, estado do Rio de Janeiro. Instituto Oswaldo Cruz, Fundação Oswaldo Cruz, Rio de Janeiro. Available at: <https://www.arca.fiocruz.br/handle/icict/372932019>.

64. Abreu FVSd, Delatorre E, Dos Santos AAC, Ferreira-de-Brito A, de Castro MG, Ribeiro IP, et al. Combination of surveillance tools reveals that Yellow Fever virus can remain in the same Atlantic Forest area at least for three transmission seasons. Memórias do Instituto Oswaldo Cruz. 2019;114.

65. Hendy A, Hernandez-Acosta E, Chaves BA, Fé NF, Valério D, Mendonça C, et al. Into the woods: Changes in mosquito community composition and presence of key vectors at increasing distances from the urban edge in urban forest parks in Manaus, Brazil. Acta Trop. 2020;206:105441. Epub 2020/03/17. doi: 10.1016/j.actatropica.2020.105441. PubMed PMID: 32173316; PubMed Central PMCID: PMCPMC7184314.

66. Hendy A, Hernandez-Acosta E, Valério D, Mendonça C, Costa ER, Júnior JTA, et al. The vertical stratification of potential bridge vectors of mosquito-borne viruses in a central Amazonian forest bordering Manaus, Brazil. Sci Rep. 2020;10(1):18254. Epub 2020/10/28. doi: 10.1038/s41598-020-75178-3. PubMed PMID: 33106507; PubMed Central PMCID: PMCPMC7589505.
