# Supplementary material for: An ecological niche model to predict the geographic distribution of Haemagogus janthinomys, Dyar, 1921 a yellow fever and Mayaro virus vector, in South America
Source: PLoS Negl Trop Dis. 2022 Jul 8;16(7):e0010564. doi: 10.1371/journal.pntd.0010564 (PMC9299311; doi:10.1371/journal.pntd.0010564)
Supplement: S2 Table — (DOCX) [file pntd.0010564.s002.docx]

| Variable Name | Description | Included | Source |
| --- | --- | --- | --- |
| BIO1 | Annual Mean Temperature | Yes | WorldClim [1] |
| BIO2 | Mean Diurnal Range (Mean of monthly (max temp - min temp)) | No |  |
| BIO3 | Isothermality (BIO2/BIO7) (×100) | No |  |
| BIO4 | Temperature Seasonality (standard deviation ×100) | No |  |
| BIO5 | Max Temperature of Warmest Month | No |  |
| BIO6 | Min Temperature of Coldest Month | No |  |
| BIO7 | Temperature Annual Range (BIO5-BIO6) | No |  |
| BIO8 | Mean Temperature of Wettest Quarter | No |  |
| BIO9 | Mean Temperature of Driest Quarter | No |  |
| BIO10 | Mean Temperature of Warmest Quarter | No |  |
| BIO11 | Mean Temperature of Coldest Quarter | No |  |
| BIO12 | Annual Precipitation | No |  |
| BIO13 | Precipitation of Wettest Month | No |  |
| BIO14 | Precipitation of Driest Month | No |  |
| BIO15 | Precipitation Seasonality (Coefficient of Variation) | Yes |  |
| BIO16 | Precipitation of Wettest Quarter | No |  |
| BIO17 | Precipitation of Driest Quarter | No |  |
| BIO18 | Precipitation of Warmest Quarter | No |  |
| BIO19 | Precipitation of Coldest Quarter | No |  |
| Enhanced vegetation index (avg.) | Average canopy greenness calculated from annual raster layers spanning the years 2000-2020 | Yes | MODIS MCD43D62-68 v6 Product [2] |
| Enhanced vegetation index (SD) | Standard deviation of canopy greenness calculated from annual raster layers spanning the years 2000-2020 | No |  |
| Slope | Values represent the angle of the downward sloping terrain from 0 (flat) to 90 degrees (vertical) | Yes | ESRI Living Atlas of the World [3] |
| Elevation | Digital elevation map of the world | Yes |  |
| Soils | Categorical variable that represents 106 soil units and 4 non-soil units | No | Food and Agricultural Organization [4] |
| Flow Direction | Flow direction from each cell to steepest downslope neighbor. Eight possible values represent direction of flow | No | Calculated from the ESRI ArcGIS Pro Spatial Analyst toolbox [5] |
| Flow Accumulation | Accumulated weight of all cells flowing into each downslope cell | No |  |
| Aspect | The direction of each cell’s slope, measured in degrees (from 0 to 360) | No |  |
| Urban and built-up proportional land cover | The percentage of each pixel covered by urban/built-up land cover, range 0-100 | Yes | MODIS v6 MCD12Q1 Land Cover Product [6] |
| Evergreen broadleaf forest proportional land cover | The percentage of each pixel covered by evergreen broadleaf forest land cover, range 0-100 | Yes |  |
| Savanna proportional land cover | The percentage of each pixel covered by savanna land cover, range 0-100 | No |  |
| Grassland proportional land cover | The percentage of each pixel covered by grassland land cover, range 0-100 | No |  |
| Categorical land cover class | Categorical | No |  |

References

1. Fick SE, Hijmans RJ. WorldClim 2: new 1km spatial resolution climate surfaces for global land areas. International Journal of Climatology. 2017;37(12):4302-15.

2. Lin Q, editor Enhanced vegetation index using Moderate Resolution Imaging Spectroradiometers. 2012 5th International Congress on Image and Signal Processing; 2012 16-18 Oct. 2012.

3. Danielson JJ, Gesch DB. Global Multi-resolution Terrain Elevation Data 2010 (GMTED2010). 2011.

4. ESRI. Soils of the world from the from the United Nations Food and Agriculture Organization. 2014.

5. ESRI. ArcGIS Desktop: Release 10.8 Redlands, CA: Environmental Systems Research Institute2019.

6. Friedl MA, Sulla-Menashe D, Tan B, Schneider A, Ramankutty N, Sibley A, et al. MODIS Collection 5 global land cover: Algorithm refinements and characterization of new datasets. Remote sensing of Environment. 2010;114(1):168-82.
