# Supplementary figures and images for: An ecological niche model to predict the geographic distribution of Haemagogus janthinomys, Dyar, 1921 a yellow fever and Mayaro virus vector, in South America
Source: PLoS Negl Trop Dis. 2022 Jul 8;16(7):e0010564. doi: 10.1371/journal.pntd.0010564 (PMC9299311; doi:10.1371/journal.pntd.0010564)

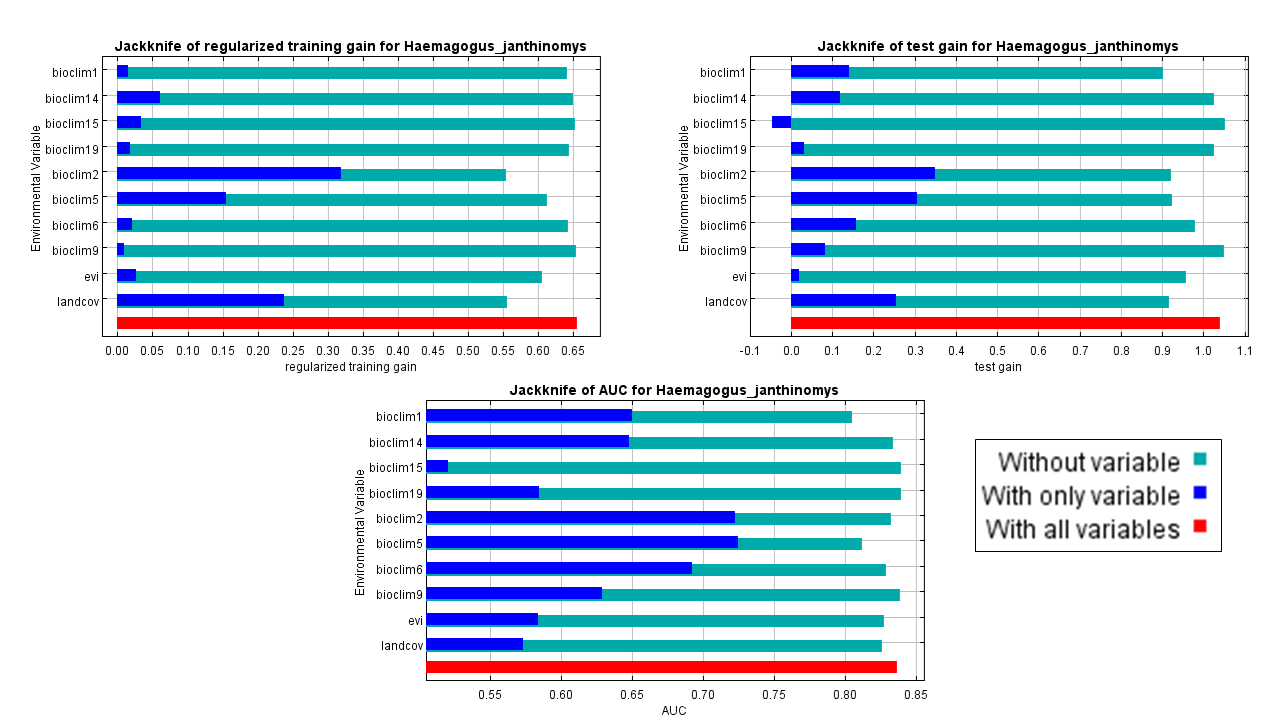

Supplement: S1 Fig — (TIF) [file pntd.0010564.s003.tif]
